# Left Ventricular and Atrial Function Analysis Following Transcatheter Edge-to-Edge Mitral Valve Repair

**DOI:** 10.3390/jcm13237282

**Published:** 2024-11-29

**Authors:** Timor Linder, Doron Sudarsky, Liza Grosman-Rimon, Jordan Rimon, Mony Shuvy, Shemy Carasso

**Affiliations:** 1 Cardiovascular Institute, Tzafon Medical Center, Poriya 1528001, Israel; timorlmegiddo@gmail.com (T.L.); dormers@gmail.com (D.S.); 2 The Azriely Faculty of Medicine, Bar-Ilan University, Zefat 1311502, Israel; 3 Wingate Institute, School of Graduate Studies, The Academic Center Levinsky-Wingate, Netanya 4290200, Israel; l.grosman.rimon@gmail.com; 4 Faculty of Health, York University, Toronto, ON M3J 1P3, Canada; jordan.rimon@gmail.com; 5 Structural Heart Service, The Jesselson Integrated Heart Center, Shaare Zedek Medical Center, Affiliated with the Faculty of Medicine, The Hebrew University, Jerusalem 9103102, Israel; monysh@szmc.org.il; 6 Non-Invasive Cardiac Imaging, Shaare Zedek Medical Center, Affiliated with the Faculty of Medicine, The Hebrew University, Jerusalem 9103102, Israel

**Keywords:** mitral regurgitation, transcatheter edge-to-edge mitral valve repair, left atrium, speckle tracking echocardiography

## Abstract

**Background:** Conventional echocardiography used to assess volumes of the left ventricle (LV) and left atrium (LA) along with mitral regurgitation grade is routine in studies before and after transcatheter edge-to-edge mitral valve repair (Mitral TEER). Previous studies focus on LV parameter changes and comparison of the functions before and a few months following Mitral TEER implantation, as well as LA reverse remodeling, by assessing LV volumes. However, less is known regarding LA strain changes in the early phase after the procedure. The objective of the study was to assess the effect of Mitral TEER on LA strain early after TEER procedure. **Methods:** The retrospective study included 44 patients who underwent Mitral TEER. LA strain and volumes were evaluated by speckle tracking echocardiography at the baseline and 24–48 h following the procedure. Demographic, echocardiographic, and clinical characteristics were obtained and statistically analyzed. **Results:** LA global longitudinal strain (GLS) reservoir improved significantly (from 12.2 ± 7 to 14.7 ± 6.4, *p* = 0.0079) after Mitral TEER. Significant improvements were also seen in LA volumes (LA maximal and minimal volume), which reduced by 17% and 22.5% respectively. LV GLS was significantly changed (from −9.8% to −12.8%, *p* < 0.0001) following Mitral TEER, whereas LV stroke volume was not significantly different between the baseline and post-Mitral TEER (*p* = 0.7798). **Conclusions:** After successful Mitral TEER, there was a very early improvement in LA function. Two-dimensional speckle tracking echocardiography may contribute to our understanding of LA functional changes immediately post-procedure.

## 1. Introduction

Transcatheter edge-to-edge mitral valve repair (Mitral TEER) has emerged as an alternative treatment option for patients who are not eligible for cardiac surgery. Although Mitral TEER is less effective at reducing mitral regurgitation (MR) compared to surgery, the procedure demonstrated superior safety and similar clinical improvement [1].

Prior to and after Mitral TEER, conventional echocardiography is routinely used for assessment of the left ventricle and atrium volumes, along with mitral regurgitation grade [2,3,4]. Speckle tracking echocardiography, a novel imaging modality for assessing myocardial mechanics such as strain, has been shown to have clinical prognostic value in patients with MR [5,6]. In a recent study by the COAPT group, improved left atrial (LA) strain at the 6-month follow-up was associated with subsequently lower rates of the composite endpoints of all-cause mortality or heart failure hospitalization, both after TEER and GDMT alone [7]. Lower pre-TEER left ventricular (LV) global longitudinal strain was shown to be associated with a worse prognosis after TEER [8]. However, LA and LV strain are not routinely assessed before and after Mitral TEER procedure

Studies have shown that after successful Mitral TEER, changes in echocardiographic parameters are associated with improved outcomes, including left ventricular reverse remodeling (LVRR), defined as a 10% reduction in left ventricular end-diastolic volume (LVEDV), which is associated with LA dimension reduction and improvement in heart failure symptoms and survival [9,10]. Indeed, speckle tracking echocardiography evaluating both myocardial deformation (percentage of change in myocardial length and strain) and volumes throughout the cardiac cycle [11] may improve the assessment of the left atrial remodeling and function before and after Mitral TEER.

Previous studies focused on LA reverse remodeling, following Mitral TEER by assessing left ventricle and atrium volumes [2,4,12]. However, less is known regarding LA and LV strain changes in the early phase after the procedure. In a recent review of the LA role in secondary MR [13], it was suggested that LA function improvement after transcatheter MV repair may be associated with improved prognosis, and needs to be studied. The objective of the study was to assess the effect of Mitral TEER on LA and LV strain early after TEER procedure.

## 2. Materials and Methods

### 2.1. Study Design

An observational retrospective study was conducted, using patients’ charts and imaging studies from Poriya Medical Center’s (PMC) ongoing Mitral TEER registry.

The study was approved by the Ethical Review Board of Poriya Medical Center and complies with the guidelines of the Declaration of Helsinki. All patients included in this study provided informed consent.

### 2.2. Patient Population

**Figure 1:**
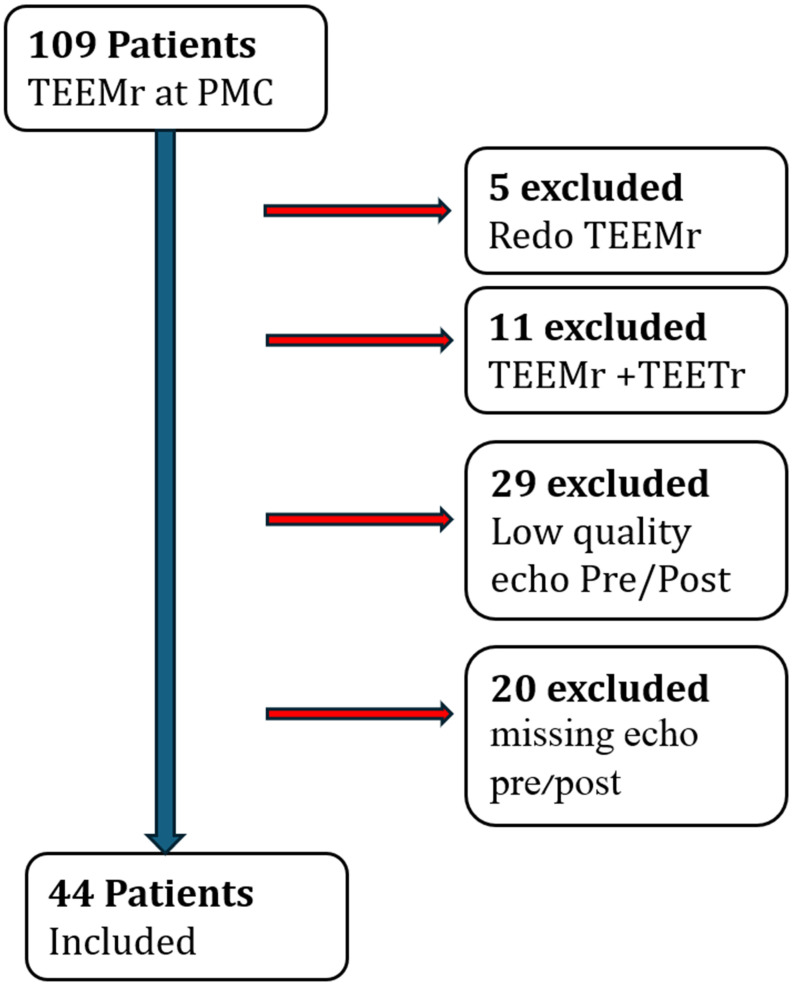
Inclusion–Exclusion flow chart. TTEMr, mitral edge-to-edge repair, TEETr, tricuspid edge-to-edge repair, PMC, Poriya Medical Center.

In total, 109 patients had undergone Mitral TEER between March 2015 and February 2022 at PMC. The patients were assessed by a multidisciplinary heart team and were referred to Mitral TEER due to high surgical risk. All patients were diagnosed with ≥moderate-severe MR and remained symptomatic despite optimal medical therapy. For patients who underwent a repeated Mitral TEER, we included only the first procedure in the analysis. Patients who underwent a combined procedure of mitral and tricuspid clipping had either missing or low image quality (inability to track LV or LA segments, foreshortened LA views—in either pre-procedure or discharge studies), and those with missing images were excluded, leaving 44 patients available for analysis (Figure 1). Patient follow-up for all-cause mortality was 511 days (333–793, 95% Confidence interval).

### 2.3. Procedure

Mitral TEER was performed per clinical indications after assessment by a cardiologist. All procedures were performed with the MitraClip^®^ device (Abbott Vascular, Santa Clara, CA, USA), according to a standard technique [1]. The procedure was performed under general anesthesia, with the imaging guidance of fluoroscopy and 2-dimensional (2D) and 3-dimensional (3D) TEE.

### 2.4. Two-Dimensional and Doppler Echocardiography

2D-TTE was performed prior to the Mitral TEER and again at discharge (performed 1.7 ± 1.8 days post-procedure). Studies were conducted by two experienced sonographers on various machine systems (GE Vivid E9, GE Medical Systems, Milwaukee, WI, USA; Philips Epiq7CVx, Philips Medical System, Andover, MA, USA; Siemens Acuson SC2000 PRIME, Siemens Medical Solutions, Malvern, PA, USA), applying a common acquisition protocol. The two interpreting echocardiographers were not blind to the clinical diagnosis and Mitral TEER or post-procedural course.

### 2.5. Tissue Strain Echocardiography

The LA and LV measurements were retrospectively analyzed, using the software eSie VVI, version 3.01.168b.180216, Siemens Medical System, Mountain View, CA, USA. Acquired 4-chamber, 3 chamber, and 2-chamber cine images were uploaded to the software. Myocardial mechanics analyses were performed by a single operator (TL) and supervised by a guiding echocardiographer (SC) that reviewed and approved all analyses. The endocardial region of interest was manually marked. The cardiac cycle was determined by the onset of R wave for LA strain and volume curves. Biplane LA function was assessed, including reservoir parameters (maximal and minimal volumes, maximal strain), as half of the patients were in atrial fibrillation lacking LA booster function. Biplane LV volumes were assessed in a similar fashion using 4-chamber and 2-chamber views (bi plane volumes). LV longitudinal strain was measured from the Acquired 4-chamber, 3-chamber, and 2-chamber views.

### 2.6. Statistical Analysis

The data were analyzed using SPSS software, version 18.0 (SPSS Inc., Chicago, IL, USA). Continuous data with normal distribution were reported as the mean and one standard deviation (SD) and compared using dependent samples *t*-tests. Continuous variables without a normal distribution were reported as medians and interquartile ranges and were analyzed using a Mann–Whitney U test. Categorical variables were reported as absolute numbers and percentages and were compared using the Chi-Square tests. A 2-sided *p*-value of ≤0.05 was considered statistically significant.

Sample size power calculations: 45 patients were needed to detect a 3%-point change in the strain with a standard deviation (SD) of 5% points of strain in pre- and post-studies. A similar sample size was calculated for detection of a 30 mL change in volumes (SD 50 mL).

## 3. Results

In total, 44 patients who underwent Mitral TEER procedure were included in the analysis. Their baseline characteristics are shown in Table 1. All the patients had multiple comorbidities including coronary risk factors, 75% had coronary artery disease, and a third had chronic lung disease. Their average EuroScore II risk was 11.1% (95% CI 6.8–15.4%).

### 3.1. TEER Procedure Results and 2D-Doppler Echocardiography

Mitral TEER procedures were performed without complications in all patients. Early morality (48 h of procedure) occurred in one patient, related to sepsis. Total mortality was found in 12 patients at a median time of 222 days from procedure (73–648, confidence interval). Data regarding mode of death were not available for most of the patients.

Following Mitral TEER, the effective regurgitant orifice area decreased significantly, while mitral valve mean pressure gradient was higher compared to the baseline (Table 2). Intraprocedural hemodynamic measurements showed a decrease in LA peak V wave from 30 ± 13 to 21 ± 9 mmHg (*p* = 0.0023). At 3 months follow-up, the NYHA functional class dropped by 1 point compared to assessment at the baseline.

**Figure 2:**
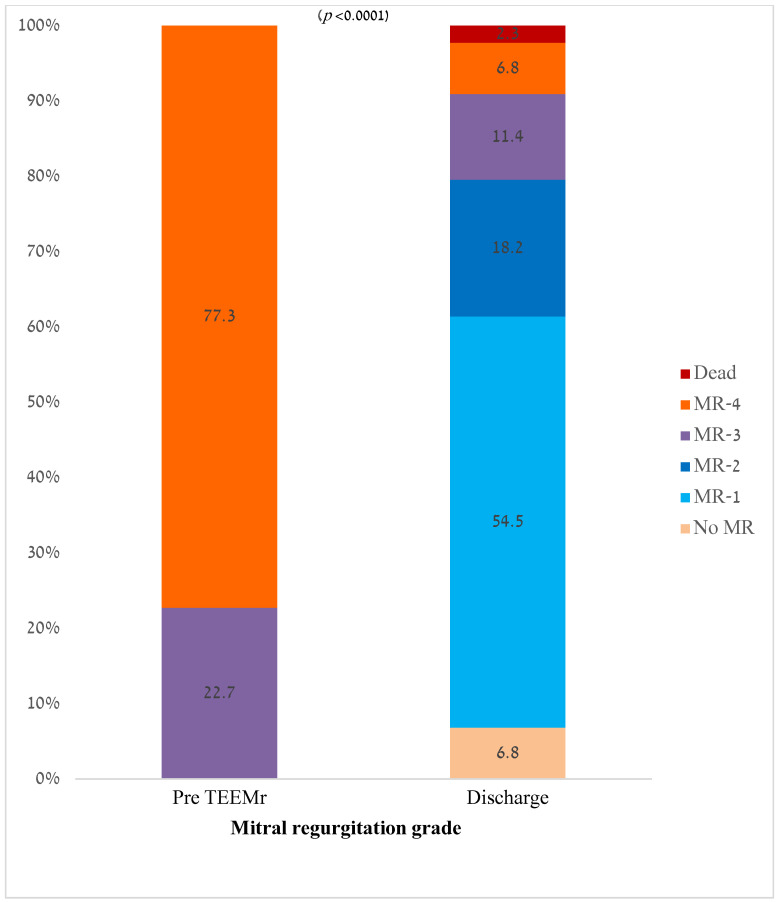
Mitral regurgitation grade at baseline and pre-discharge.

Pre-procedural MR severity and residual MR severity at discharge are presented in Figure 1. Following Mitral TEER, MR severity at discharge significantly decreased compared to pre-procedure values (1 (1–2) vs. 4 (4–4), *p* < 0.0001). While before Mitral TEER procedure all the patients had significant MR (MR-3 or MR-4), approximately 80% of patients were discharged with MR-2 or less. There was no relation between MR grade decrease and the MR mechanism (primary vs secondary, *p* = 0.825) (Figure 2).

### 3.2. Myocardial Mechanics—LA and LV Strain and Volumes

Left ventricular and left atrial myocardial strain and volume assessments, at baseline and prior to discharge 24–48 h post-Mitral TEER, are presented in Table 3. Patients with secondary MR had similar strains and volumes at the baseline. LV end diastolic and end systolic volume decreased by 11% and 16%, respectively, while LV stroke volume did not change. LV GLS showed a significant 30% improvement after Mitral TEER (*p* < 0.001). Similarly, LA maximal and minimal volumes were reduced by 17% and 22.5% respectively, while LA reservoir stroke volume did not significantly change (*p* = 0.8441). At discharge, LA GLS reservoir improved by 25% after Mitral TEER (14.7 ± 6.4 and 12.2 ± 7, *p* = 0.0079). The extent of changes in volume and strain did not correlate with the MR mechanism. Improved LA reservoir strain > 7% points was found to be associated with improved survival (OR 5.18 95% CI 1.17–22.97, *p* = 0.031).

## 4. Discussion

Our study investigated the early changes in LA strain parameters immediately after Mitral TEER. The main finding of the study is that following successful Mitral TEER, both left chambers decrease in size and improve their mechanics—while their respective stroke volumes remain unchanged. These changes occurred very early after TEER.

Mitral regurgitation results in LA and LV remodeling, which are associated with a poor prognosis and an increased risk of cardiac death [14,15]. Following successful Mitral TEER, LV reverse remodeling is associated with LV strain changes, LA dimension reduction and improvement in heart failure symptoms and survival [16,17,18]. While most studies focused on LV strain following Mitral TEER [16,17,18,19], only a few studies have reported the LA strain changes, and importantly the immediate changes were not reported previously.

### 4.1. LA Function Post Mitral TEER

Previous studies have demonstrated that after successful reduction of degenerative MR severity, LA peak positive strain decreased significantly in patients with normal/high baseline strain, when treated with either Mitral TEER or mitral valve surgical repair at 12 months follow-up [13,20]. In comparison, in our study, LA GLS reservoir was higher at discharge compared to pre-Mitral TEER values. However, while all the patients, in the former study, suffered from primary MR, in our study the majority of the patients (70.5%) suffered from secondary MR with very different pathophysiology, clinical course, and prognosis [19]. Additionally, the researchers found a significant decline in LA strain that was more marked in surgical patients than in Mitral TEER patients, while we studied only patients who underwent Mitral TEER. Surgical MVR requires cutting the LA open and is in many cases accompanied with a MAZE procedure for treatment of atrial fibrillation, both of which may hamper LA function. However, another study evaluating LA remodeling reported that compared to the baseline, LA reservoir strain improved significantly after Mitral TEER, along with significant improvements in three-dimensional (3D) minimum LA volume index and maximum LA volume index [19]. It is important to note that in that study the follow-up was performed at 12 months post-procedure and 8% of the patients included in the study suffered from primary MR. In our study, we assessed LA strain indices early within post-procedural index hospitalization, and more than ¼ of the patients suffered from either primary or mixed etiology MR.

The LA reservoir phase is essential for LV filling because the energy stored by the LA during ventricular systole is released after mitral valve opening, greatly contributing to LV stroke volume, a mechanism that depends on LA compliance, which is altered in chronic MR patients [21]. Reducing MR severity may decrease preload and LA expansion, improving LA compliance. In our study, we found a significant early improvement in LA GLS reservoir at discharge (after 24–48 h). Additionally, compared with the baseline, LA minimum and maximum volumes were reduced without a significant change in LA stroke volume, further demonstrating the early changes in LA after TEER. As a result of the MR reduction, the backward flow into the LA decreases, subsequently decreasing LA minimal and maximal volumes. Since both minimum and maximum LA volumes are lower post-Mitral TEER, the LA stroke volume remains the same. Similar results regarding LA volumes were described by other Mitral TEER studies [20,22].

### 4.2. LV Function Post Mitral TEER

LV function was assessed after Mitral TEER implantation in a few studies. Following Mitral TEER, a previous study reported no change at all in LV GLS one year after the procedure [23]. Furthermore, one study described a decrease in LV GLS compared to baseline [16]. In this study, LV and LA longitudinal strain worsened only in patients that had an increase in N-terminal pro brain natriuretic peptide (NT-proBNP) after Mitral TEER, suggesting an increase of myocardial wall stress. Cimino et al. concluded that MR reduction results in improvement of LV GLS, only if reverse remodeling occurred. Those patients with LV reverse remodeling and LV GLS improvement had better baseline parameters [17]. Another study showed LV and RV strain improvement after clip implantation as well as lower post-procedural LV strain values in patients with worse preexisting RV function [18]. Six-month 3D LV GLS was correlated significantly with improvements in NYHA functional class and 6-minute walk distance. Early improvements, at discharge, were seen in 2D LV longitudinal strain and in 3D LV longitudinal strain. Our study demonstrated similar results and a very early improvement in LV GLS. Post-clip implantation, the volume-overload is reduced and therefore wall stress is ameliorated, manifested by better LV function [19]. The changes in LV may also explain the early LA remodeling, since during systole and early diastole, LA expansion and shortening on the longitudinal axis are primarily influenced by movement of the valvular plane. Therefore, LA stain may reflect the longitudinal LV systolic function in addition to LA intrinsic function [22]. Along with the strain changes, we found no significant change in the overall stroke volume of the LV, although there was a significant decrease in MR degree and LA volume. This may be explained by the MR reduction, which causes an increase in the forward stroke volume with a concomitant decrease in the backward flow into the LA whereas total stroke volume remains unchanged significantly.

Chamber size, wall stress, and pressures are interrelated as depicted in the LaPlace theorem from 1806 and Woods application in 1892 [24]. Generally, wall stress increases as the chamber size (radius) increases [25]. As previously demonstrated, myocardial wall stress increases oxygen consumption [26,27,28]. Thus, improved myocardial physiology, with potentially reduced oxygen demand for the ejection of similar volumes at rest, may explain the improvement in dyspnea and effort tolerance in patients with severe MR after successful TEER.

While we attribute these strain and volume changes directly to the TEER procedure, they may well be attributed to acute post-procedural adjustments in drug therapy, including diuretics, inotropes (up or down titration, for either), and utilization of mechanical support devices. Having the second echo performed pre-discharge after the post-procedural stormy time may have decreased the likelihood of these effects but not eliminated them. The durability of the mechanical LA and LV remodeling needs also to be evaluated, and more importantly study their association with clinical outcomes, such as mortality, congestive heart symptoms, and general well-being. We plan to address these in a multicenter study.

### 4.3. Study Limitations

Since the study was conducted in a single center, we had a relatively small number of patients available for analysis, which may have introduced a selection bias. Therefore, a larger sample size is necessary for validating our results. Another limitation of our study is that LA strain measurement requires a good echocardiographic imaging, and we had to exclude from our study patients with low echocardiographic quality and this may affect the significance of our results. Both sinus rhythm and atrial fibrillation patients were included in the study and further investigation regarding LA strain differences between those two groups is needed, after clip implantation. Similarly, a quarter of our patients had right atrial pacing, which may have influenced left atrial mechanics and hemodynamics.

## 5. Conclusions

A successful Mitral TEER leads to early LV and LA reverse remodeling, manifesting as a functional improvement, which can be detected very early after the procedure by the LV and LA strain study. Those immediate changes may further contribute to our understanding of the conditions associated with an improved functional and clinical course following Mitral TEER. A large multi-center study is needed to confirm the findings of this study and associate them with long-standing clinical outcomes.

## Figures and Tables

**Table 1:** Baseline characteristics.

		*n* = 44	**%**
**Male sex (*n*, %)**		26	59.1%
**Age (years)**		75.07 ± 8.69	
**Body Mass Index (kg/m^2^)**		27.01 ± 3.91	
**Smoking (Past and Present)**		18	40.9%
**Hypertension (*n*, %)**		37	84.1%
**Dyslipidemia (*n*, %)**		36	81.8%
**Diabetes Mellitus (*n*, %)**		23	52.3%
**COPD/OSA (*n*, %)**		15	34%
**CKD (GFR < 60 mL/min/mr^2^)**		8	18%
**Atrial Fibrillation**		22	50%
**RA pacing (dual chamber, CRT)**		11	25%
**Coronary Artery Disease (*n*, %)**		33	75%
**Previous MI (*n*, %)**		29	65.9%
**Previous CVA/TIA (*n*, %)**		5	11.4%
**Previous CABG (*n*, %)**		7	15.9%
**MR mechanism (*n*, %)**	**Primary** **Secondary** **Mixed**	10/44	22.7%
		31/44	70.5%
		3/44	6.8%

MI, myocardial infarction; CVA, cerebrovascular accident; TIA, transient ischemic attack; COPD, Chronic obstructive pulmonary disease; CABG, Coronary artery bypass graft surgery, OSA—obstructive sleep apnea, RA, right atrial, CRT, cardiac resynchronization therapy.

**Table 2:** Pre and Post TEER 2D-Doppler Echocardiography.

Echocardiographic and Clinical Parameters			
	Pre-Implantation	Post-Implantation	*p*-Value
**Left Ventricle End Diastolic Diameter (mm)**	62 ± 9	60.6 ± 9.2	0.1817
**Left Ventricle End Systolic Diameter (mm)**	50 ± 10.1	49.4 ± 11	0.7041
**Mitral Valve EROA (cm^2^)**	0.47 ± 0.27	0.22 ± 0.13	0.0005
**Mitral Valve Regurgitant Volume (mL)**	62.3 ± 29.3	23.8 ± 17.7	0.0003
**Mitral Valve Mean Pressure Gradient (mmHg)**	1.8 ± 1.6	3.6 ± 2.1	<0.0001
**RVSP (mmHg)**	49.3 ± 14.8	40.1 ± 11.8	0.0002
**Reversal pulmonary veins flow pattern**	27/42 (64.3%)	4/42 (9.5%)	<0.0001
NYHA FC *	3 (2–3)	2 (1–2)	<0.0001

* Changes in NYHA are between baseline and 3 months follow-up and reported as median and interquartile range (IQR). EROA, effective regurgitant orifice area; RVSP, right ventricular systolic pressure; NYHA FC, New York Heart Association functional class.

**Table 3:** LA and LV Myocardial Mechanics at baseline and after Mitral TEER.

	Pre-Implantation	Post-Implantation	*p*-Value
**Heart rate (BPM)**	79 ± 17	74 ± 16	0.0965
**Sinus Rhythm (*n*, %)**	22 (50)	24 (55)	0.5719
**LV EDV (mL)**	169 ± 65	151 ± 99	0.0075
**LVESV (mL)**	118 ± 58	99 ± 54	0.0005
**LV stroke volume (mL)**	51 ± 20	53 ± 22	0.7798
**LVEF (%)**	33 ± 12	36 ± 11	0.0410
**LV GLS (%)**	−9.8 ± 4.4	−12.8 ± 4	<0.0001
**LA V_max_ (mL)**	124 ± 47	103 ± 38	0.0008
**LA V_min_ (mL)**	89 ± 42	69 ± 34	0.0002
**LA SV (mL)**	35 ± 16	35 ± 13	0.8441
**LA EF Reservoir (%)**	23 ± 14	36 ± 13	0.0113
**LA GLS Reservoir (%)**	12 ± 7	15 ± 6	0.0079
**LA Conduit volume (mL)**	16 ± 21	18 ± 20	0.7461

LV, left ventricle; LVEF, Left ventricle ejection fraction; GLS, global longitudinal strain; SV, stroke volume; V_max_, volume maximum; V_min_, volume minimum.

## Data Availability

The original contributions presented in the study are included in the article, further inquiries can be directed to the corresponding author.
